# Differential modulation of pulmonary caspases: Is this the key to *Ureaplasma*-driven chronic inflammation?

**DOI:** 10.1371/journal.pone.0216569

**Published:** 2019-05-08

**Authors:** Christine Silwedel, Markus Fehrholz, Christian P. Speer, Katharina C. Ruf, Steffi Manig, Kirsten Glaser

**Affiliations:** 1 University Children´s Hospital, University of Wuerzburg, Wuerzburg, Germany; 2 Institute of Hygiene and Microbiology, University of Wuerzburg, Wuerzburg, Germany; Miami University, UNITED STATES

## Abstract

Although accepted agents in chorioamnionitis and preterm birth, the role of *Ureaplasma* species (spp.) in inflammation-driven morbidities of prematurity, including the development of bronchopulmonary dysplasia, remains controversial. To add to scarce *in vitro* data addressing the pro-inflammatory capacity of *Ureaplasma* spp., pulmonary epithelial-like A549 cells and human pulmonary microvascular endothelial cells (HPMEC) were incubated with *Ureaplasma (U*.*) urealyticum*, *U*. *parvum*, and *Escherichia coli* lipopolysaccharide (LPS). *Ureaplasma* isolates down-regulated caspase mRNA levels in A549 cells (caspase 8: *p*<0.001, 9: *p*<0.001, vs. broth), while increasing caspase protein expression, enzyme activity, and cell death in HPMEC (active caspase 3: *p*<0.05, caspase 8: *p*<0.05, active caspase 9: *p*<0.05, viability: *p*<0.05). LPS, contrarily, induced caspase mRNA expression in HPMEC (caspase 3: *p*<0.01, 4: *p*<0.001, 5: *p*<0.001, 8: *p*<0.001, vs. control), but not in A549 cells, and did not affect enzyme activity or protein levels in either cell line. LPS, but neither *Ureaplasma* isolate, enhanced mRNA expression of pro-inflammatory interleukin (*IL)-6* in both A549 (*p*<0.05, vs. control) and HPMEC (*p*<0.001) as well as tumor necrosis factor-α (*p*<0.01), *IL-1β* (*p*<0.001), and *IL-8* (*p*<0.05) in HPMEC. We are therefore the first to demonstrate a differential modulation of pulmonary caspases by *Ureaplasma* spp. *in vitro*. *Ureaplasma*-driven enhanced protein expression and activity of caspases in pulmonary endothelial cells result in cell death and may cause structural damage. Down-regulated caspase mRNA in pulmonary epithelial cells, contrarily, may indicate *Ureaplasma*-induced inhibition of apoptosis and prevent effective immune responses. Both may ultimately contribute to chronic *Ureaplasma* colonization and long-term pulmonary inflammation.

## Introduction

*Ureaplasma* species (spp.) commonly colonize the adult urogenital tract and are generally considered of low virulence [1]. Transmission from mother to infant is frequent and can occur *in utero*, intrapartum, or postpartum [1]. Intraamniotic *Ureaplasma* infection is an accepted risk factor for chorioamnionitis and premature birth [2–4], and *Ureaplasma* spp. are known to cause sepsis, meningitis, and pneumonia in neonates [5–8] as well as severe invasive infections in immunocompromised adults such as lung transplant patients [9]. *Ureaplasma* spp. can be detected in the respiratory tract in 65% of preterm infants < 26 weeks of gestation [10]. Fetal or neonatal respiratory tract colonization with *Ureaplasma* spp. has been associated with bronchopulmonary inflammation and altered lung development, which may ultimately culminate in chronic lung diseases such as bronchopulmonary dysplasia (BPD) in preterm infants [11–13]. Inflammation is considered a key factor in the multifactorial pathogenesis of BPD development [13, 14]. Animal models support a potential causality between *Ureaplasma* spp. and development of BPD, demonstrating pulmonary inflammation accompanied by structural lung tissue impairment upon fetal *Ureaplasma* exposure [15, 16]. Clinical studies, however, are contradictory [17], and *in vitro* data on the pro-inflammatory capacity of *Ureaplasma* spp. are generally scarce.

In pulmonary inflammation, lung epithelial and endothelial cells both deserve attention. They usually maintain intrapulmonary homeostasis as well as an immunological balance [18, 19]. Pulmonary epithelial cells maintain the air-blood barrier and comprise alveolar type I and II cells [20]. While type I cells primarily enable gas exchange, type II cells produce surfactant and are crucial for tissue repair [19]. Opposed to epithelial cells, lung endothelial cells are more permeable [21] and contribute to inflammatory processes by signal transduction and initiation of inflammatory cell migration into the alveolar space [19, 22].

Inflammation is classically understood to be initiated by early pro-inflammatory cytokines, including tumor necrosis factor (TNF)-α, interleukin (IL)-1β, IL-6, and IL-8 [23]. However, recent studies have suggested an interlinkage of apoptosis and inflammation, proposing a crucial role for caspases in both [24, 25]. Whereas caspases 3, 8, and 9 are among those primarily involved in apoptosis, caspases 4 and 5 mediate inflammatory responses and pyroptosis as an inflammatory form of cell death [26, 27]. Regulation of caspase activity is complex and involves the transcription and translation processes as well as specific cleavage and activation of synthesized inactive caspase proenzymes [27].

We could recently demonstrate caspase modulation and a pro-apoptotic capacity of *Ureaplasma* spp. in human brain microvascular endothelial cells (HBMEC) [28]. In the present study, we addressed *Ureaplasma*-induced caspase expression as well as cytokine responses in the well described epithelial type II cell-like line A549 and human pulmonary microvascular endothelial cell line HPMEC-ST1.6R (HPMEC) [19, 29–31].

## Materials and methods

### Bacterial strains and culture conditions

*Ureaplasma (U*.*) urealyticum* serovar 8 and *U*. *parvum* serovar 3 were obtained from the American Tissue Culture Collection (ATCC, Manassas, VA; serovar 8: ATCC 27618, serovar 3: ATCC 27815). Isolates were cultured in *in-house* modified 10-B medium [32] (referred to as “broth”), containing 82% pleuropneumonia-like organism medium (Becton, Dickinson & Company, Franklin Lakes, NJ), 10% heat-inactivated horse serum (v/v), 1% urea (w/v) and 0.002% phenol red (w/v) (all: Sigma-Aldrich, St. Louis, CA), adjusted to pH 6.5. An endotoxin level < 0.06 EU/mL broth was verified using ToxinSensor Endotoxin Detection System (GenScript, Piscataway, NJ). Serial 10-fold dilutions were incubated for 18–20 h to achieve titers of 1×10^9^−1×10^10^ color-changing units (CCU)/mL of viable cells.

### Eukaryotic cells and culture conditions

A549 cells (ATCC CRM-CCL-185) were cultured in DMEM (Sigma-Aldrich) supplemented with 10% fetal bovine serum (Gibco, Thermo Fisher Scientific, Waltham, MA), 100 U/mL penicillin, and 100 μg/mL streptomycin (Sigma-Aldrich). HPMEC-ST1.6R [31] were cultured in M199 Medium (Biochrom, Merck, Darmstadt, Germany) supplemented with 10% fetal bovine serum (Gibco), 2 mM L-glutamine (Biochrom), 5000 U/mL heparin (Biochrom), 5 μg/mL endothelial cell growth supplement (Omnilab, Bremen, Germany), 100 U/mL penicillin, and 100 μg/mL streptomycin. Cells were incubated in their respective growth media at 37°C in a humidified atmosphere with 5% CO_2_.

For stimulation assays, 3.5×10^5^ A549 cells and 2.5×10^5^ HPMEC per well were seeded in uncoated six well plates (Greiner, Frickenhausen, Germany). 24 h later, cells were stimulated as indicated in 1 mL fresh growth medium without antibiotics.

### Stimulation assays

*Ureaplasma* suspensions of 10^9^−10^10^ CCU/mL were concentrated by centrifugation, and 10^9^−10^10^ CCU in 250 μl broth were added to each well. CCU were determined by 10-fold titration, as described elsewhere [33], and corresponding genome equivalents were identified, amounting to 5×10^7^−6×10^8^ copy numbers/mL (Institute of Medical Microbiology and Hospital Hygiene, Duesseldorf, Germany) [34]. Viability was verified by simultaneous culture on selective agar plates (medco Diagnostika GmbH, Ottobrunn, Germany). Lipopolysaccharide (LPS, *Escherichia coli* serotype 055:B5, Sigma-Aldrich) was added to a subgroup of cells at a concentration of 100 ng/mL. All doses had been determined by preliminary assays analogous to previous approaches [33, 35–40]. According to results of preliminary time kinetic experiments [35], exposure times of 4 and 30 h were chosen for RNA analysis, whereas flow cytometry was performed after 24 h. Experiments were repeated at least 3 times (n ≥ 3). Unstimulated cells served as negative controls. To adjust for potential confounding effects of *Ureaplasma* medium, cells exposed to *Ureaplasma* isolates were additionally compared to broth control and results were considered relevant if comparisons to both negative controls showed *p* values < 0.05.

### RNA extraction and RT-PCR

For RNA extraction, cells were treated as indicated and total RNA was isolated using NucleoSpin RNA Kit (Macherey-Nagel, Dueren, Germany). For quantification of total RNA, a Qubit 2.0 Fluorometer (Thermo Fisher Scientific) was employed. Total RNA was eluted in 60 μL RNase-free H_2_O (Macherey-Nagel) and stored at -80°C until reverse transcription. For RT-PCR, 0.5–1 μg of total RNA was reverse transcribed using High Capacity cDNA Reverse Transcription Kit (Thermo Fisher Scientific). First strand cDNA was diluted 1 to 10 with deionized, nuclease-free H_2_O (Sigma-Aldrich) and stored at -20°C upon analysis.

### Quantitative real time RT-PCR (qRT-PCR)

For quantitative detection of mRNA, 10 μL of diluted first strand cDNA were analyzed in duplicates of 25 μL reactions using 12.5 μL iTaq Universal SYBR Green Supermix (Bio-Rad Laboratories, Hercules, CA), 0.5 μL deionized H_2_O, and 1 μL of a 10 μM solution of forward and reverse primers (Sigma-Aldrich) as indicated in Table 1. Primers were designed using PerlPrimer software v1.1.20 [41]. Amplicons were designed to be 100–200 bp in size. A BLAST search was performed for every primer to confirm specificity with E values < 1. At least one primer of each pair spanned an intron/exon boundary to ensure mRNA-specific amplification. PCRs were performed on an Applied Biosystems 7500 Real-Time PCR System (Thermo Fisher Scientific), using a 2-step PCR protocol after an initial denaturation at 95°C for 10 min with 40 cycles of 95°C for 15 s and 60°C for 1 min. A melt curve analysis was performed at the end of every run to verify single PCR products. Levels of mRNA were normalized to those of glyceraldehyde-3-phosphate dehydrogenase (GAPDH). Mean fold changes in mRNA expression were calculated by the ΔΔC_T_ method by Livak and Schmittgen [42].

**Table 1:** Primers for qRT-PCR.

Gene symbol	Sequence accession #	Orientation	Sequence [5’ to 3’]	Amplicon length [bp]
*CASP3*	NM_004346.3	forward	CATTGAGACAGACAGTGG	108
		reverse	TCGCCAAGAATAATAACCAG	
*CASP4*	NM_001225.3	forward	GTTTGACCATCTGCCTCC	126
		reverse	CGCTGACTCCATATCCCT	
*CASP5*	NM_004347.3	forward	CTTTCTGTTCTTCAACACCA	143
		reverse	ATGATTTCTGTACCTTCCGA	
*CASP8*	NM_001228.4	forward	CTGATTCAGAGGAGCAACCC	200
		reverse	GAATATCATCGCCTCGAGGAC	
*CASP9*	NM_001229.4	forward	CCATATCTAGTTTGCCCACAC	183
		reverse	GAAACAGCATTAGCGACCCT	
*CXCL8*	NM_000584.3	forward	CAGTGCATAAAGACATACTCC	198
		reverse	TTTATGAATTCTCAGCCCTC	
*GAPDH*	NM_002046.5	forward	CCATGGAGAAGGCTGGGG	195
		reverse	CAAAGTTGTCATGGATGACC	
*IL1B*	NM_000576.2	forward	TTCATTGCTCAAGTGTCTG	128
		reverse	GCACTTCATCTGTTTAGGG	
*IL6*	NM_000600.4	forward	AACAAATTCGGTACATCCTC	167
		reverse	AAGTCTCCTCATTGAATCCA	
*TNF*	NM_000594.3	forward	CAGCCTCTTCTCCTTCCT	188
		reverse	GGGTTTGCTACAACATGG	

### Flow cytometry

Harvested cells were separated by centrifugation and stained with Fixable Viability Dye eFluor 780 (eBioScience, San Diego, CA). Centrifugation and resuspension in Phosphate Buffered Saline (PBS, Sigma-Aldrich) were followed by fixation (fixation buffer, BioLegend). After another centrifugation step, cells were permeabilized (permeabilization wash buffer, BioLegend) and stained with antibodies to cleaved caspase 3 (Alexa Fluor 647 conjugated, rabbit, Cell Signaling Technology, Danvers, MA), caspase 8 (unconjugated, mouse, Abcam, Cambridge, UK), and cleaved caspase 9 (PE conjugated, rabbit, Cell Signaling Technology). To detect the unconjugated caspase 8 antibody, cells were once more separated by centrifugation and stained with an Alexa Fluor 405 conjugated secondary antibody (goat anti-mouse, Life Technologies, Thermo Fisher Scientific). After centrifugation and resuspension of the cells in PBS containing 1% human serum (Biochrom), samples were read on a FACSCanto II flow cytometer (BD Biosciences, San Jose, CA). At least 10,000 events were acquired and analyzed with FACSDiva v6.1.3 software (BD Biosciences). Events were gated via forward and side scatter, and doublets were excluded employing a SSC-width versus FSC-area dot plot (the gating strategy is described in S1 Fig). For viability assessment, all events were included and the percentage of viability dye positive cells, considered dead, was determined.

### Statistical analysis

Data were analyzed by a one way ANOVA with Tukey’s multiple comparisons test using Prism 6 software (GraphPad Software, San Diego, CA). The significance threshold for *p* values was set at < 0.05. Data are shown as means ± standard deviation (SD).

## Results

### *Ureaplasma-*driven cell death in pulmonary epithelial and endothelial cells

**Fig 1:**
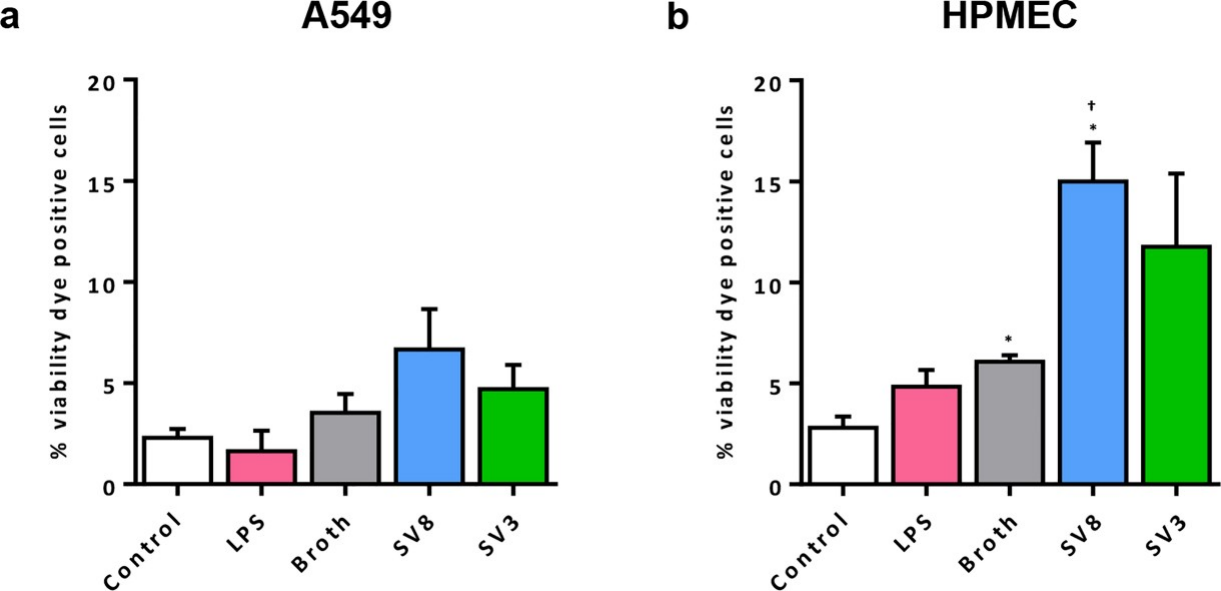
*Ureaplasma*-driven cell death in pulmonary epithelial and endothelial cells. Following a 24 h stimulation period, numbers of dead cells were determined for different conditions using flow cytometry and a viability dye staining dead cells. Results for A549 cells are shown in (a), whereas (b) depicts the percentage of dead HPMEC. Data are presented as means ± SD and were obtained from n = 3 individual experiments. Cells stimulated with LPS were compared vs. control, cells exposed to *Ureaplasma* spp. vs. control and vs. broth. * *p* < 0.05 compared to untreated controls; † *p* < 0.05 compared to cells treated with broth. SV8: *Ureaplasma urealyticum* serovar 8, SV3: *Ureaplasma parvum* serovar 3.

Flow cytometry was used to identify viability dye positive, dead cells. In A549 cells, we generally counted low numbers of dead cells, without any significant influences on cell viability yielded by *Ureaplasma* spp. (Fig 1A). Exposure of HPMEC to both *Ureaplasma* isolates, however, caused a distinct increase in numbers of dead cells after 24 h (Fig 1B), which was significant for serovar 8 (2.5 ± 0.3-fold, *p* = 0.0347, vs. broth). Broth itself induced a mild increase in dead cells. LPS did not affect viability of A549 cells or HPMEC (Fig 1).

### *Ureaplasma*-driven caspase responses in pulmonary epithelial cells

**Fig 2:**
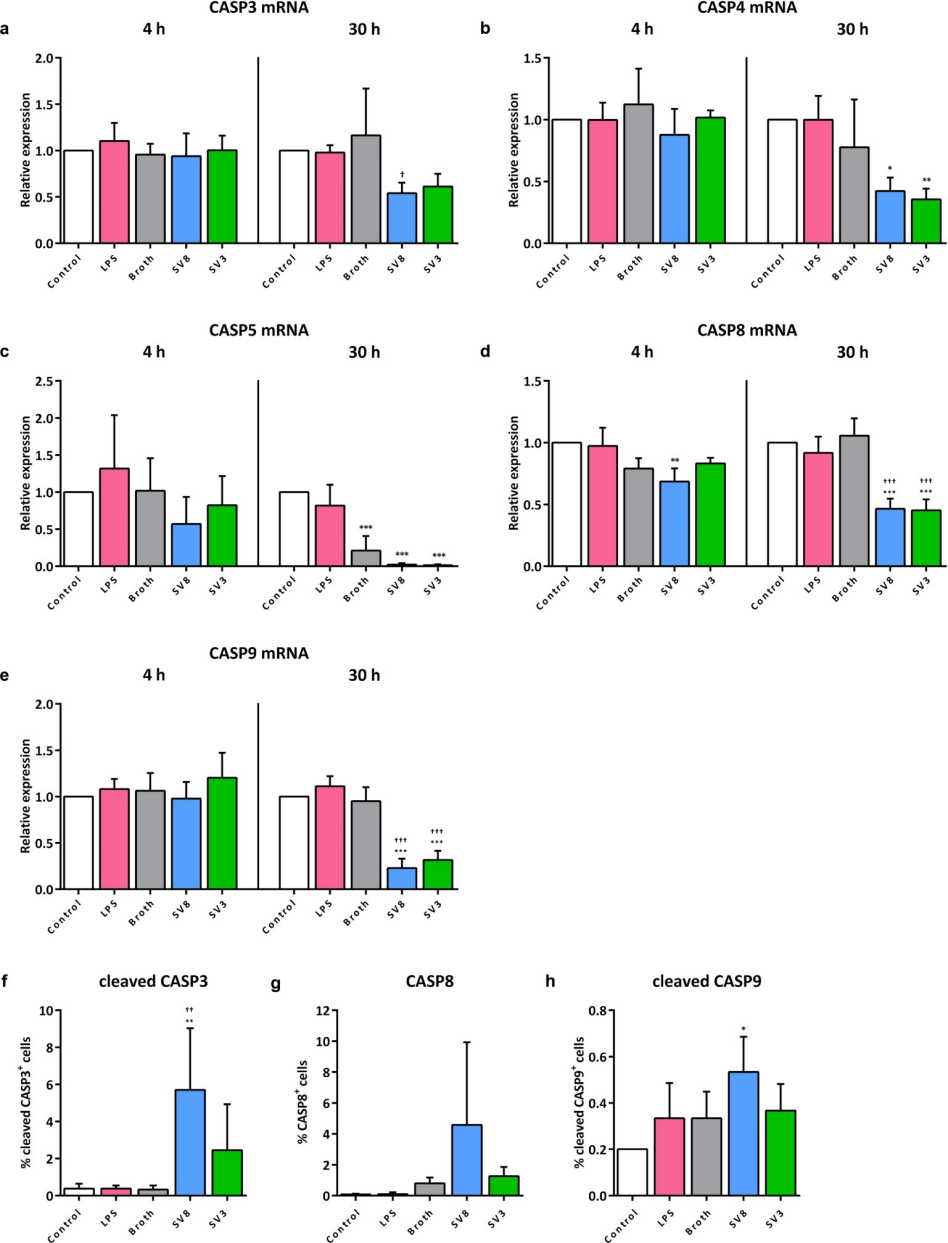
*Ureaplasma*-driven caspase mRNA and protein responses in A549 cells. After 4 and 30 h stimulation of A549 cells, mRNA levels of caspase (CASP) 3 (a), caspase 4 (b), caspase 5 (c), caspase 8 (d), and caspase 9 (e) were assessed via qRT-PCR, and relative expression was calculated using the ΔΔC_T_ method. After 24 h stimulation, the percentage of viable, active caspase 3 (f), caspase 8 (g), and active caspase 9 (h) positive A549 cells was determined by flow cytometry (the respective gating strategy is illustrated in S1 Fig). Data are shown as means ± SD and were obtained from n ≥ 3 independent experiments. Cells stimulated with LPS were compared vs. control, cells exposed to *Ureaplasma* isolates vs. control and vs. broth. * *p* < 0.05, ** *p* < 0.01, and *** *p* < 0.001 compared to untreated controls; † *p* < 0.05, †† *p* < 0.01, and ††† *p* < 0.001 compared to cells treated with broth. SV8: *Ureaplasma urealyticum* serovar 8, SV3: *Ureaplasma parvum* serovar 3.

For caspases 3, 4, and 5, we observed a trend towards lower mRNA levels following 30 h *Ureaplasma* exposure of A549 cells, which mostly did not reach statistical significance (caspase 3, serovar 8: 0.5 ± 0.1-fold, *p* = 0.0442, serovar 3: 0.5 ± 0.1-fold, *p* = 0.0828; caspase 4, serovar 8: 0.5 ± 0.1-fold, *p* = 0.2332, serovar 3: 0.5 ± 0.1-fold, *p* = 0.1228; caspase 5, serovar 8: 0.1 ± 0.09-fold, *p* = 0.5481, serovar 3: 0.07 ± 0.06-fold, *p* = 0.5106, vs. broth) (Fig 2A–2C). *Ureaplasma* stimulation of A549 cells for 30 h resulted in a significant down-regulation of mRNA expression of caspase 8 (serovar 8: 0.4 ± 0.1-fold, *p* < 0.001, serovar 3: 0.4 ± 0.1-fold, *p* < 0.001, vs. broth) (Fig 2D). An even more distinct reduction was observable for caspase 9 mRNA upon a 30 h exposure to serovar 8 (0.2 ± 0.1-fold, *p* < 0.001) and serovar 3 (0.3 ± 0.1-fold, *p* < 0.001, vs. broth) (Fig 2E). Broth itself had mild suppressive effects on caspase 5 (Fig 2C). A short-term *Ureaplasma* exposure for 4 h as well as stimulation of A549 cells with LPS for 4 or 30 h did not significantly influence mRNA levels of caspases 3, 4, 5, 8, or 9 (Fig 2A–2E).

Flow cytometry revealed slightly higher levels of active (cleaved) caspase 3 upon 24 h exposure to serovar 8 (17.5 ± 10.2-fold, *p* = 0.0075, vs. broth, Fig 2F). *Ureaplasma* exposure did not affect caspase 8 protein abundance or caspase 9 activity in A549 cells, and neither did LPS evoke any responses on caspase protein or activity levels (Fig 2F–2H).

### *Ureaplasma*-driven caspase responses in pulmonary endothelial cells

**Fig 3:**
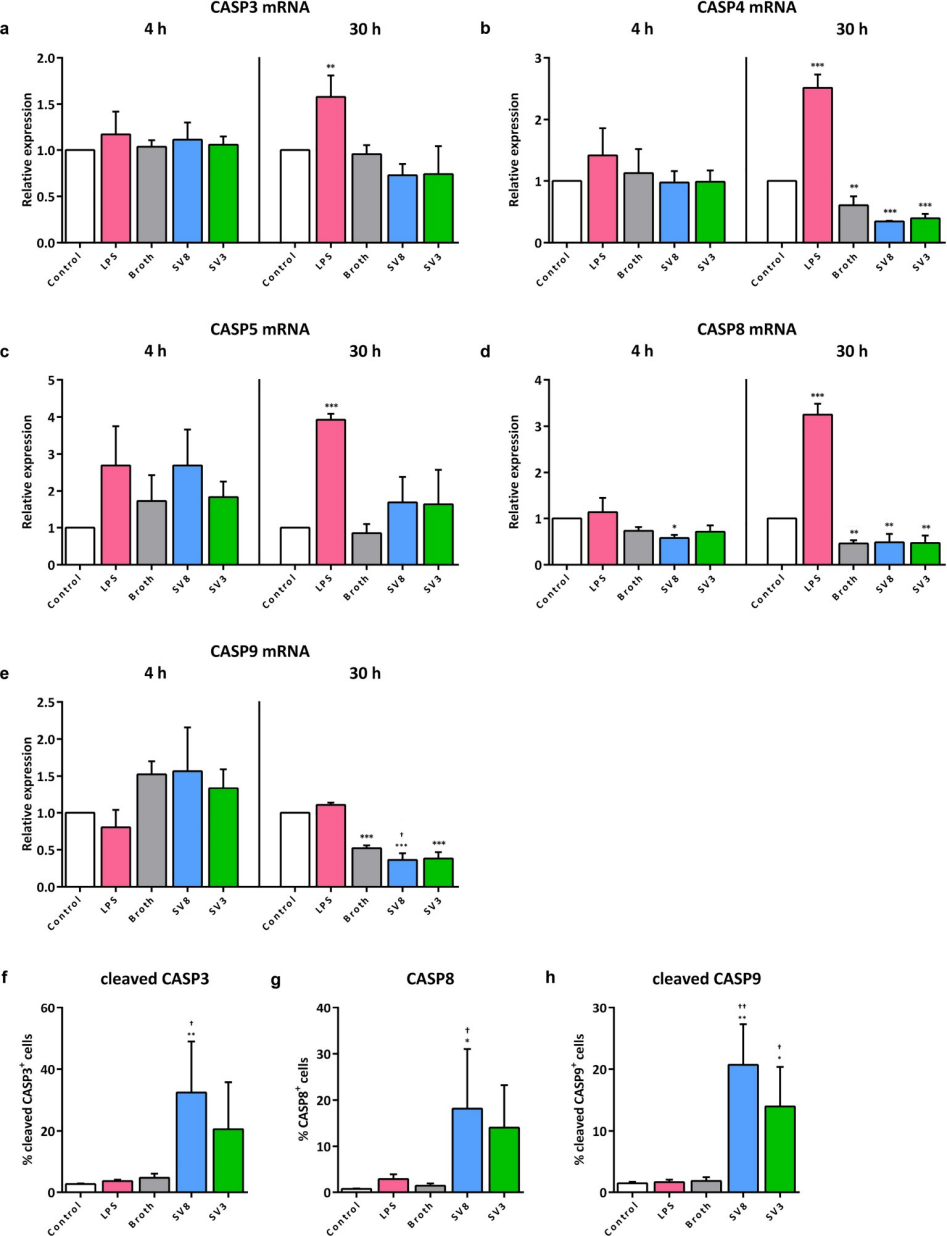
*Ureaplasma*-driven caspase mRNA and protein responses in HPMEC. Following an incubation period of 4 and 30 h, mRNA levels of caspase (CASP) 3 (a), caspase 4 (b), caspase 5 (c), caspase 8 (d), and caspase 9 (e) were obtained via qRT-PCR, and relative expression was calculated using the ΔΔC_T_ method. The percentage of viable, active caspase 3 (f), caspase 8 (g), and active caspase 9 (h) positive HPMEC was determined by flow cytometry after 24 h stimulation (the respective gating strategy is illustrated in S1 Fig). Data are presented as means ± SD from n ≥ 3 independent experiments. Cells stimulated with LPS were compared vs. control, *Ureaplasma* exposed cells vs. control and vs. broth. * *p* < 0.05, ** *p* < 0.01, and *** *p* < 0.001 compared to untreated controls; † *p* < 0.05, and †† *p* < 0.01 compared to cells treated with broth. SV8: *Ureaplasma urealyticum* serovar 8, SV3: *Ureaplasma parvum* serovar 3.

With the exception of a mild reduction of caspase 9 mRNA upon 30 h of serovar 8 exposure (0.7 ± 0.2-fold, *p* = 0.0374, vs. broth), *Ureaplasma* isolates did not influence caspase mRNA levels in HPMEC (Fig 3A–3E). Variations in comparison to unstimulated control cells were not significant compared to broth control, since broth itself had moderate and inconsistent effects on caspase mRNA expression. LPS stimulation of HPMEC for 4 h did not evoke any significant mRNA variances for caspases 3, 4, 5, 8, and 9 (Fig 3A–3E). After 30 h of LPS exposure, however, we could observe significantly increased mRNA levels for caspase 3 (1.6 ± 0.2-fold, *p* = 0.0086, vs. control, Fig 3A), caspase 4 (2.5 ± 0.2-fold, *p* < 0.001, Fig 3B), caspase 5 (3.9 ± 0.2-fold, *p* < 0.001, Fig 3C), and caspase 8 (3.3 ± 0.2-fold, *p* < 0.001, Fig 3D).

Flow cytometry revealed an *Ureaplasma*-induced significant increase of positive cells for cleaved caspase 3 (serovar 8: 6.9 ± 3.5-fold, *p* = 0.0104, vs. broth), caspase 8 (serovar 8: 12.9 ± 9.2-fold, *p* = 0.0315), and cleaved caspase 9 (serovar 8: 11.3 ± 3.6-fold, *p* = 0.0016; serovar 3: 7.6 ± 3.5-fold, *p* = 0.0303) (Fig 3F–3H), with serovar 8 tending to have slightly stronger effects than serovar 3. LPS had no significant influences on caspase 3, 8, or 9 protein expression or enzyme activity in HPMEC, respectively (Fig 3F–3H).

### *Ureaplasma*-driven cytokine responses in pulmonary epithelial and endothelial cells

**Fig 4:**
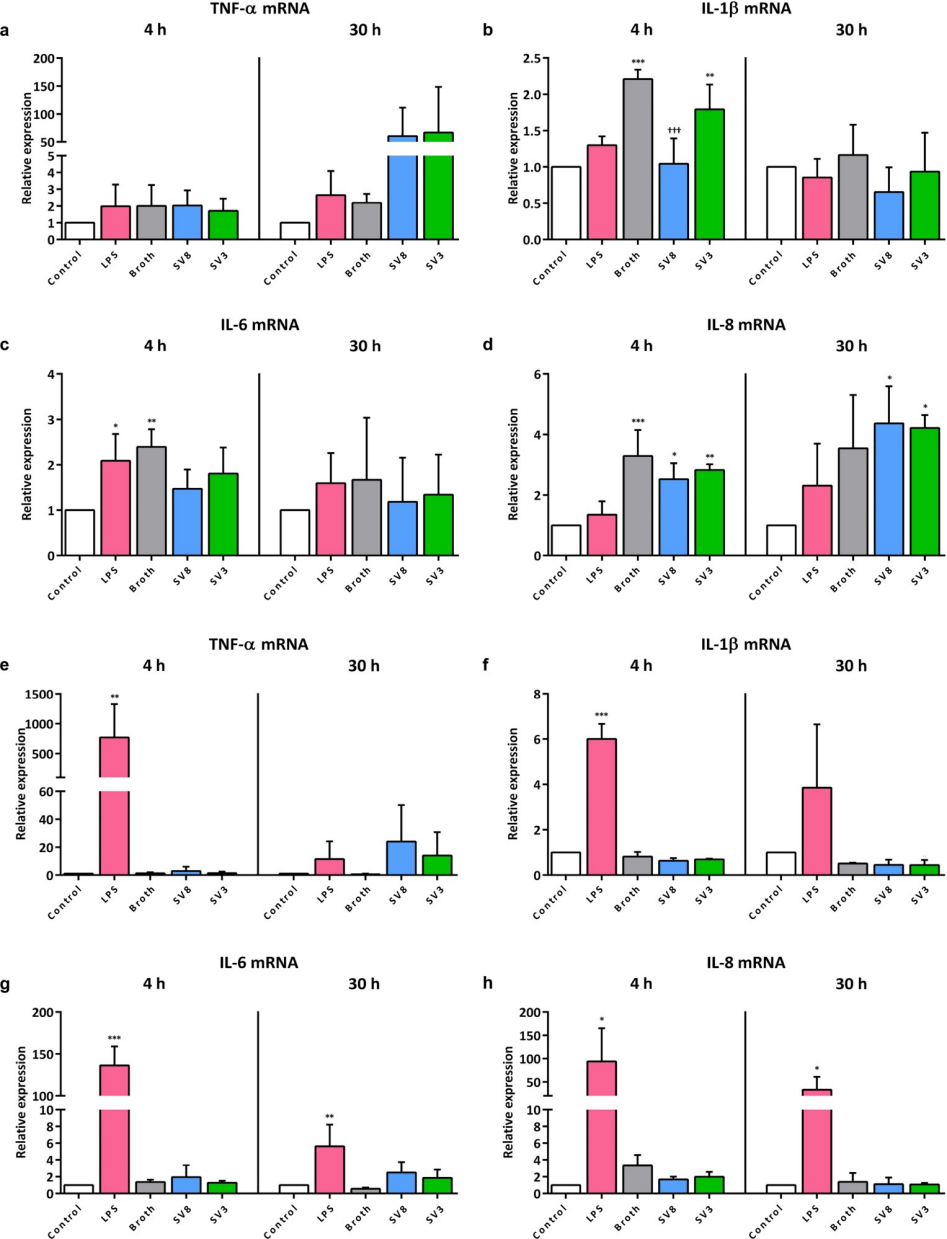
*Ureaplasma*-driven mRNA expression of pro-inflammatory cytokines in A549 cells and HPMEC. In A549 cells, mRNA levels of TNF-α (a), IL-1β (b), IL-6 (c), and IL-8 (d) were assessed via qRT-PCR after 4 and 30 h stimulation. Similarly, mRNA expression in HPMEC was determined for TNF-α (e), IL-1β (f), IL-6 (g), and IL-8 (h). Data are presented as means ± SD from n ≥ 3 independent experiments. LPS stimulated cells were compared vs. control, cells exposed to *Ureaplasma* isolates vs. control and vs. broth. * *p* < 0.05, ** *p* < 0.01, and *** *p* < 0.001 compared to untreated controls; ††† *p* < 0.001 compared to cells treated with broth. SV8: *Ureaplasma urealyticum* serovar 8, SV3: *Ureaplasma parvum* serovar 3.

In A549 cells, no significant *TNF-α*, *IL-1β*, *IL-6*, and *IL-8* mRNA responses were measurable upon *Ureaplasma* exposure if compared to broth and control, since broth itself had a certain cytokine-inducing effect (Fig 4A–4D). Exposure of A549 cells to LPS for 4 h induced a significant increase of *IL-6* mRNA (2.1 ± 0.6-fold, *p* = 0.0430, vs. control), whereas LPS did not influence mRNA levels of *TNF-α*, *IL-1β*, and *IL-8*, regardless of the duration of stimulation (Fig 4A–4D).

In HPMEC, *Ureaplasma* spp. similarly did not have any significant effects on mRNA levels of all given cytokines (Fig 4E–4H). LPS was able to significantly induce mRNA expression of *TNF-α* (4 h: 770 ± 557-fold, *p* = 0.0097, vs. control), *IL-1β* (4 h: 6.0 ± 0.7-fold, *p* < 0.001), *IL-6* (4 h: 136 ± 22.8-fold, *p* < 0.001, 30 h: 5.6 ± 2.6-fold, *p* = 0.0048), and *IL-8* (4 h: 94.0 ± 71.1-fold, *p* = 0.0140, 30 h: 33.0 ± 27.8-fold, *p* = 0.0307) (Fig 4E–4H).

### Caspase and cytokine responses upon co-stimulation of pulmonary epithelial and endothelial cells

Co-stimulation of A549 cells or HPMEC with LPS and *Ureaplasma* isolates did not significantly aggravate effects observed after mono-stimulation with one or the other stimulus regarding caspase 3, 4, 5, 8, and 9 mRNA expression, cleaved caspase 3, caspase 8, and cleaved caspase 9 protein levels, or *TNF-α*, *IL-1β*, *IL-6*, and *IL-8* mRNA levels (S2 Fig–S4 Fig).

## Discussion

This is the first *in vitro* study assessing influences of *Ureaplasma* spp. on pulmonary epi- and endothelial cells. Results indicate *Ureaplasma*-driven suppression of apoptosis in pulmonary epithelial cells and a simultaneous pro-apoptotic capacity of *Ureaplasma* spp. in pulmonary endothelial cells. Influencing caspase levels and activity may be one of the mechanisms *Ureaplasma* spp. employ to relevantly interfere with immunological processes.

**Fig 5:**
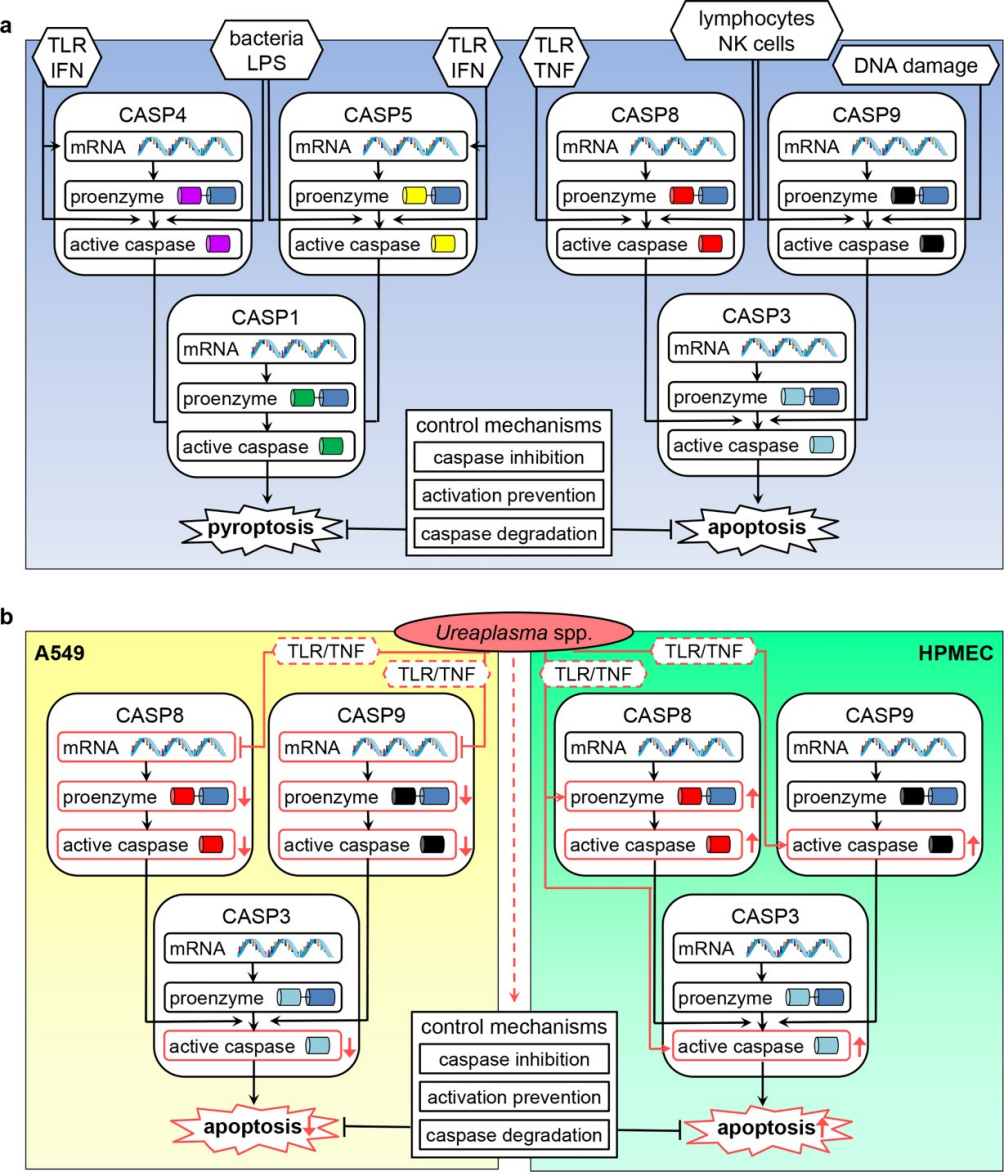
Cascade of caspase activation and potential pathways influenced by *Ureaplasma* spp. Simplified scheme (a) depicting caspase activation processes. Multiple trigger factors can initiate apoptosis and pyroptosis. Under engagement of several additional proteins not mentioned here, initiator caspases 4 and 5 for pyroptosis or 8 and 9 for apoptosis are produced and activated. These subsequently activate effector caspases 1 or 3. Caspases are activated by cleavage or dimerization, often followed by a maturational process. Control mechanisms confine programmed cell death [26, 27, 43–45]. The potential influence of *Ureaplasma* spp. on apoptosis according to our results is illustrated in (b). Pathways affected by *Ureaplasma* isolates are marked in red. In A549 cells, *Ureaplasma*-triggered reduction of caspase 8 and 9 mRNA may result in an absent increase in protein production and active caspases. Ultimately, effector caspase 3 remains unactivated and apoptosis is impaired. In HPMEC, *Ureaplasma* spp. seem to enhance caspase 8 protein and caspase 9 activity. Both caspases may subsequently activate effector caspase 3 and induce apoptosis. It remains to be determined if the caspase 3 activation observed is directly due to *Ureaplasma* or a consequence of initiator caspase activation. Hypothesized underlying mechanisms *Ureaplasma* isolates may engage are indicated with a dashed line. Ⱶ inhibit / down-regulate; ← activate / up-regulate. CASP: caspase; IFN: interferon; LPS: lipopolysaccharide; NK: natural killer; TLR: toll-like receptor; TNF: tumor necrosis factor. Illustrations: https://smart.servier.com/.

We could demonstrate that exposure of A549 cells to *Ureaplasma* spp. resulted in a distinct reduction of caspase mRNA and lacking protein responses (Fig 2), whereas in HPMEC, *Ureaplasma* isolates did not influence mRNA expression, but promoted caspase activation and protein increase (Fig 3). These divergent results regarding mRNA and protein levels can be explained by the meticulous multistep process in caspase protein production and activation (Fig 5A) [27, 43]. The *Ureaplasma*-induced caspase mRNA suppression we could demonstrate in A549 cells may result in impaired protein production. Consequently, we observed no relevant pathogen-induced caspase protein increase in A549 cells. Caspase mRNA synthesis in HPMEC appeared mostly unaffected by *Ureaplasma*-stimulation. These cells, consequently, were able to exercise an unconfined caspase response regarding protein abundance and enzyme activity.

To fully appreciate the implications of *Ureaplasma*-driven caspase modulation, the specific functions of individual caspases have to be considered. Caspases 4 and 5 are inflammatory caspases which, via caspase 1, trigger pyroptosis of macrophages, epithelial cells, and lymphocytes as an effective defense mechanism against intracellular pathogens [26] (Fig 5A). Caspases 3, 8, and 9, on the contrary, are primarily apoptotic caspases, with caspase 3 being a key effector, and caspase 8 and 9 initiating apoptosis [26] (Fig 5A).

*Ureaplasma*-associated down-regulation of caspase 8 and 9 mRNA in A549 cells and consecutively absent protein responses may therefore suppress apoptosis (Fig 5B). Apoptosis as an immune defense mechanism is particularly relevant in elimination of intracellular pathogens [45]. Given the ability of *Ureaplasma* spp. to invade host cells [46, 47], down-regulation of caspases may function as an escape mechanism *Ureaplasma* spp. use to prevent eradication. In line with this hypothesis, we did not observe an impact of *Ureaplasma* isolates on cell viability in A549 cells (Fig 1). Impaired apoptosis and subsequently reduced pathogen eradication may ultimately facilitate chronic *Ureaplasma* colonization. *Ureaplasma* spp. are known to cause long-lasting pulmonary infections [48], and BPD-development in preterm infants, to name one example, has been associated with long-term respiratory tract *Ureaplasma* colonization in particular [49]. Our findings may therefore be of considerable clinical relevance.

Whereas several intracellular pathogens appear to be able to suppress apoptosis, the capacity for down-regulation or inactivation of caspases has been recognized for only a few of them [50, 51]. Our data are the first to indicate *Ureaplasma*-associated down-regulation of apoptotic caspases, which may be relevant not only in pulmonary cells.

*Ureaplasma*-driven enhanced caspase 8 protein levels and enzyme activity of caspases 3 and 9 in HPMEC (Figs 3 and 5B), in contrast, provide first *in vitro* evidence for a pro-apoptotic effect of *Ureaplasma* spp. in pulmonary endothelial cells. We could confirm *Ureaplasma*-driven cell death in HPMEC by flow cytometric viability assessment (Fig 1B).

Apoptosis has a dual role with both beneficial as well as harmful effects. It contributes to immune defense and repair processes on the one hand, but facilitates tissue damage on the other [45]. A meticulous balance between growth and apoptosis is essential for normal lung development *in utero* as well as after birth [52]. *Ureaplasma*-driven increase or inhibition of apoptosis may disturb this physiological balance and may begin to impair structural lung development even prenatally, with severe implications as, for example, seen in BPD pathogenesis. Indeed, early structural lung tissue impairment such as pulmonary fibrosis could be demonstrated in *Ureaplasma*-infected preterm infants [53], and increased apoptotic activity was demonstrated within the lungs of preterm infants having developed BPD [54].

Underlying mechanisms *Ureaplasma* spp. use to modulate caspases remain to be determined. *Ureaplasma* spp. are known to engage toll-like receptor (TLR) signaling and induce TNF-α [33, 55]. TLR as well as TNF-α are relevant initiators of apoptosis and inflammatory cell death [45] (Fig 5A). Another contributing factor may be intracellular *Ureaplasma* invasion with subsequent ammonia production and pH shift. *Ureaplasma* spp. can cause fatal hyperammonemia in lung transplant patients [56], but data regarding the influence of ammonia or pH on apoptosis and caspases are inconclusive [57–59]. Last but not least, *Ureaplasma* spp. might interfere with inhibition or degradation of caspases (Fig 5).

*Ureaplasma* isolates did not relevantly modulate inflammatory responses in this study. We found only non-significantly reduced inflammatory caspase levels in A549 cells (Fig 2), and, opposed to LPS, *Ureaplasma* spp. did not evoke distinct cytokine reactions in both pulmonary cell lines. This is in line with data from animal studies describing only mild pulmonary inflammatory reactions upon *Ureaplasma* exposure [48].

With this study, we describe distinct differences in the inflammatory responses of pulmonary epithelial and endothelial cells (Figs 1–4). Alveolar epithelial cells fulfill certain immunological functions and interact with, but do not belong to professional immunological cells [18, 60]. This possibly reduces their ability to effectively target infections. Other studies also reported insignificant cytokine induction in A549 cells [61, 62], and pathogen persistence is predominantly described in epithelial cells [63, 64]. Contrarily, opposed to earlier perceptions, pulmonary endothelial cells are increasingly regarded relevant in immune recruitment and production of pro-inflammatory proteins [65, 66]. This is reflected by our observation of *Ureaplasma-* or LPS-induced caspase and cytokine enhancements in HPMEC. Of note, we could recently demonstrate *Ureaplasma*-driven apoptosis in HBMEC [28], possibly indicating a higher vulnerability of endothelial cells in general for *Ureaplasma*-induced cell death. In any case, as many studies suggest a cross-talk between epithelial and endothelial pulmonary cells [67], affection of the one is likely to bear consequences for the other.

In this study, *U*. *urealyticum* serovar 8 appeared to evoke stronger reactions than *U*. *parvum* serovar 3 (Figs 1B, 2F, 3F and 3G). It is uncertain in how far this relates to *in vivo* conditions, since literature often does not distinguish between serovars. A relevant quantity of clinically isolated *Ureaplasma* was furthermore revealed to be hybrids of different serovars [3, 68]. *U*. *urealyticum* were nonetheless reported to cause male urethritis and endometritis in pregnancy more frequently than *U*. *parvum*, and relevant genetic differences among serovars were described [69, 70]. Compared to *U*. *parvum*, more frequent horizontal gene transfer and a higher phospholipase activity, both virulence determinants, were reported in *U*. *urealyticum* [70, 71]. However, several studies did not find relevant differences between serovars and many authors conclude that *Ureaplasma* strains do no generally differ in their pathogenicity, but that host characteristics may determine individual inflammatory responses [1, 3, 70].

Our results contradict the one previous study describing apoptosis in *Ureaplasma*-exposed A549 cells [36]. These authors, however, used heat-inactivated *Ureaplasma* isolates. Although this is common practice, heat exposure may not only kill *Ureaplasma* cells, but is also likely to destroy their immunogenic surface proteins, impairing host immune responses [72]. In our experience, heat-inactivated *Ureaplasma* spp. tend to lose their pathogenicity. The use of viable *Ureaplasma* isolates is therefore advantageous and a strength of this study. It does, however, require a complex culture medium. Although we implemented growth of *Ureaplasma* isolates in yeast-free medium and furthermore ruled out endotoxin contamination, broth itself bore a certain immunogenic effect (Figs 1–4). A reason for this might be cell affection by the altered composition of A549 / HPMEC growth medium after addition of *Ureaplasma* broth. Particularly urea has been shown to be potentially cytotoxic [73] and may therefore have induced at least parts of the broth effects observed in this study. Another potential limitation of this study is the use of well-established, but adult cell lines. Although *Ureaplasma* infections may affect immunocompromised adults, preterm and term neonates are those with the highest susceptibility [9, 17]. Future studies should therefore be conducted with cell lines of neonatal origin. Furthermore, *in vitro* settings can never fully represent *in vivo* conditions, where complex interactions between numerous mediators and cell types have to be taken into consideration. In this study, we concentrated on a purposive selection of caspases and cytokines, and we can correlate mRNA levels and enzyme activity or protein levels for only a few of them. Nonetheless, we provide first evidence for *Ureaplasma*-driven, cell-type specific interference with caspases and cell death. These findings once more suggest a profound clinical relevance of *Ureaplasma* spp. and should now encourage further research.

## Conclusions

Pathogen-triggered inflammation usually evokes fierce immune reactions, condoning tissue damage, but ultimately resulting in pathogen eradication. The clinical significance of respiratory tract *Ureaplasma* colonization, however, may be based less on fulminant and temporary inflammatory reactions, but rather on chronic, subclinical infections, in which even mild inflammatory effects cause long-term sequelae. A key pathomechanism *Ureaplasma* spp. seem to employ is an interference with the caspase system. On the one hand, *Ureaplasma*-driven increases of caspase protein expression and activity in pulmonary endothelial cells may cause apoptosis and thus relevantly contribute to structural lung impairment. On the other hand, *Ureaplasma* spp. down-regulate caspase mRNA levels in pulmonary epithelial cells, thereby potentially suppressing programmed cell death as an important immune defense mechanism. Combined, these processes may facilitate chronic infections, long-term lung injury, and possibly inflammatory lung diseases such as BPD. This study provides additional evidence for the growing perception that *Ureaplasma* spp. are no innocent bystanders, but most likely much more relevant than contemplated to date.

## Supporting information

S1 Fig Gating strategy used for analysis of flow cytometry results. This diagram illustrates the gating strategy used to determine the caspase positive, viable cells depicted in Figs 2F–2H and 3F–3H as well as S2 Fig and S3 Fig. Unstimulated, stained control cells were gated via forward and side scatter, doublets were excluded, and events in the caspase positive, viability dye negative quadrant were depicted in the respective figure. CASP: caspase.
(TIF)

S2 Fig Caspase mRNA and protein responses in A549 cells upon co-stimulation. Following 4 and 30 h of co-stimulation of A549 cells, caspase mRNA levels were assessed via qRT-PCR (a-e), and relative expression was calculated using the ΔΔC_T_ method. Flow cytometry was used to determine caspase protein or activity after 24 h stimulation (f-h), the respective gating strategy is illustrated in S1 Fig. Data are shown as means ± SD and were obtained from n ≥ 3 independent experiments. # *p* < 0.05, ## *p* < 0.01, and ### *p* < 0.001 compared to cells treated with LPS; † *p* < 0.05, †† *p* < 0.01, and ††† *p* < 0.001 compared to cells treated with broth+LPS. SV8: *Ureaplasma urealyticum* serovar 8, SV3: *Ureaplasma parvum* serovar 3.
(TIF)

S3 Fig Caspase mRNA and protein responses in HPMEC upon co-stimulation. After 4 and 30 h of co-stimulation of A549 cells, caspase mRNA levels were assessed via qRT-PCR (a-e), and relative expression was calculated using the ΔΔC_T_ method. Flow cytometry was used to determine caspase protein or activity after 24 h stimulation (f-h), the respective gating strategy is illustrated in S1 Fig. Data are shown as means ± SD and were obtained from n ≥ 3 independent experiments. # *p* < 0.05 and ### *p* < 0.001 compared to cells treated with LPS; ††† *p* < 0.001 compared to cells treated with broth+LPS. SV8: *Ureaplasma urealyticum* serovar 8, SV3: *Ureaplasma parvum* serovar 3.
(TIF)

S4 Fig Pro-inflammatory cytokine responses in A549 cells and HPMEC upon co-stimulation. Cytokine mRNA levels were assessed via qRT-PCR in A549 cells (a-d) and HPMEC (e-h) following 4 and 30 h of co-stimulation. Data are presented as means ± SD from n ≥ 3 independent experiments. # *p* < 0.05 and ## *p* < 0.01 compared to cells treated with LPS; † *p* < 0.05 compared to cells treated with broth+LPS. SV8: *Ureaplasma urealyticum* serovar 8, SV3: *Ureaplasma parvum* serovar 3.
(TIF)

## Abbreviations

ATCC: American Tissue Culture Collection
BPD: bronchopulmonary dysplasia
CCU: color-changing units
FCS: fetal calf serum
HBMEC: human brain microvascular endothelial cells
HPMEC: human pulmonary microvascular endothelial cells
ICAM-1: intercellular adhesion molecule 1
IL: interleukin
LPS: lipopolysaccharide
qRT-PCR: real time quantitative reverse transcriptase polymerase chain reaction
SD: standard deviation
spp.: species
TLR: toll-like receptor
TNF-α: tumor necrosis factor-α
U.: *Ureaplasma*
