# Supplementary figures and images for: Differential modulation of pulmonary caspases: Is this the key to Ureaplasma-driven chronic inflammation?
Source: PLoS One. 2019 May 8;14(5):e0216569. doi: 10.1371/journal.pone.0216569 (PMC6506144; doi:10.1371/journal.pone.0216569)

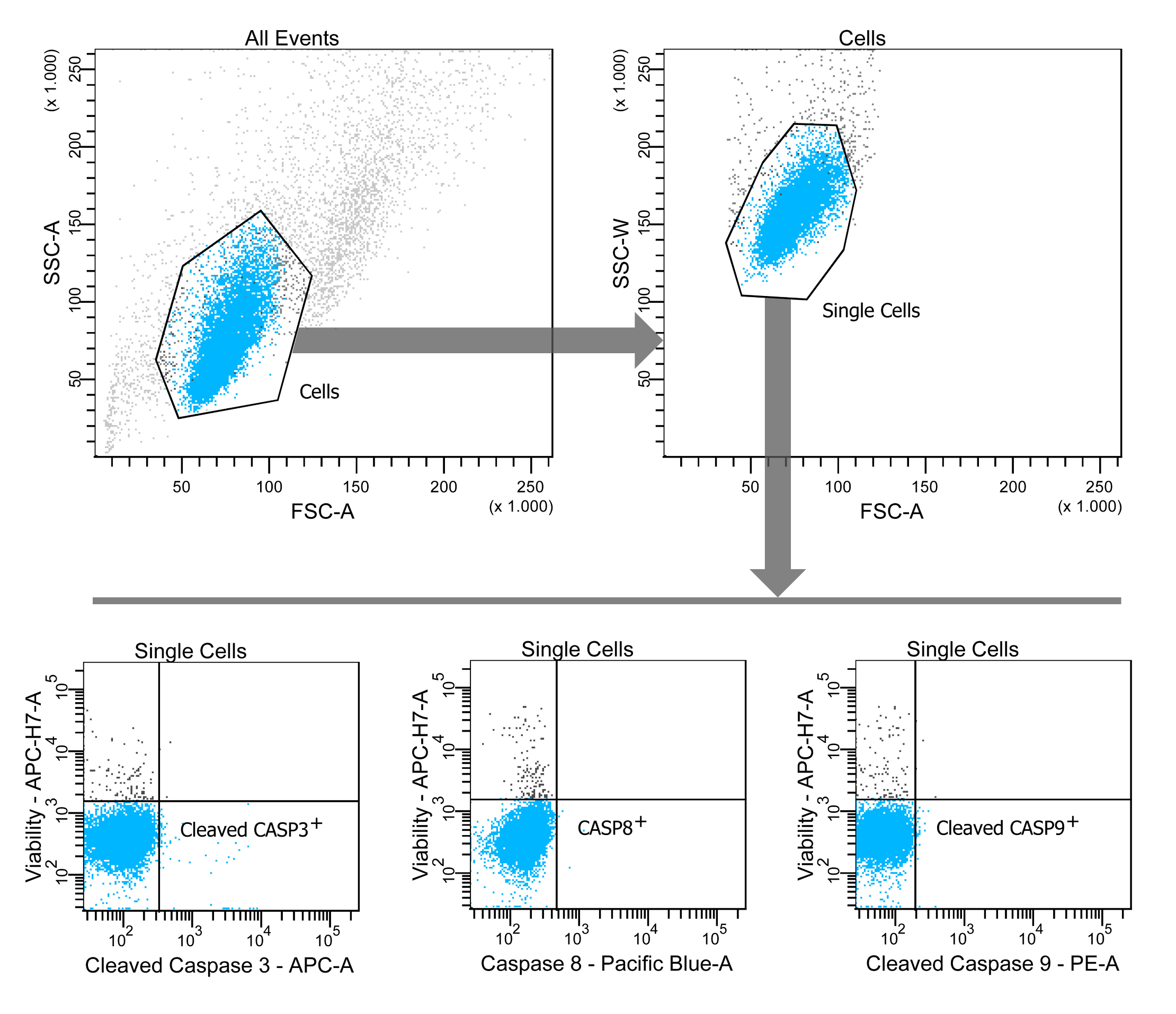

Supplement: S1 Fig — This diagram illustrates the gating strategy used to determine the caspase positive, viable cells depicted in Figs 2F–2H and 3F–3H as well as S2 Fig and S3 Fig. Unstimulated, stained control cells were gated via forward and side scatter, doublets were excluded, and events in the caspase positive, viability dye negative quadrant were depicted in the respective figure. CASP: caspase. (TIF) [file pone.0216569.s001.tif]

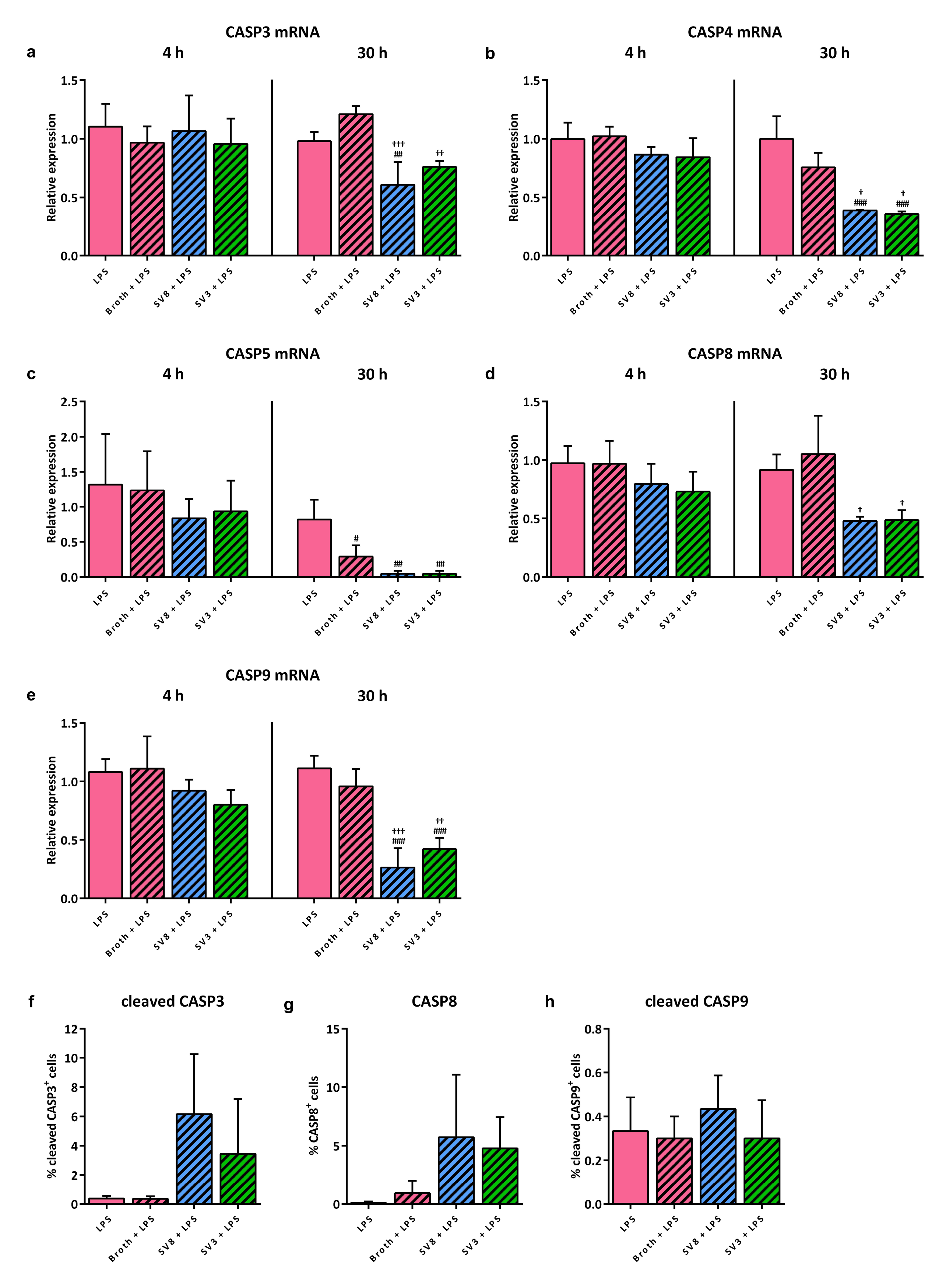

Supplement: S2 Fig — Following 4 and 30 h of co-stimulation of A549 cells, caspase mRNA levels were assessed via qRT-PCR (a-e), and relative expression was calculated using the ΔΔCT method. Flow cytometry was used to determine caspase protein or activity after 24 h stimulation (f-h), the respective gating strategy is illustrated in S1 Fig. Data are shown as means ± SD and were obtained from n ≥ 3 independent experiments. # p < 0.05, ## p < 0.01, and ### p < 0.001 compared to cells treated with LPS; † p < 0.05, †† p < 0.01, and ††† p < 0.001 compared to cells treated with broth+LPS. SV8: Ureaplasma urealyticum serovar 8, SV3: Ureaplasma parvum serovar 3. (TIF) [file pone.0216569.s002.tif]

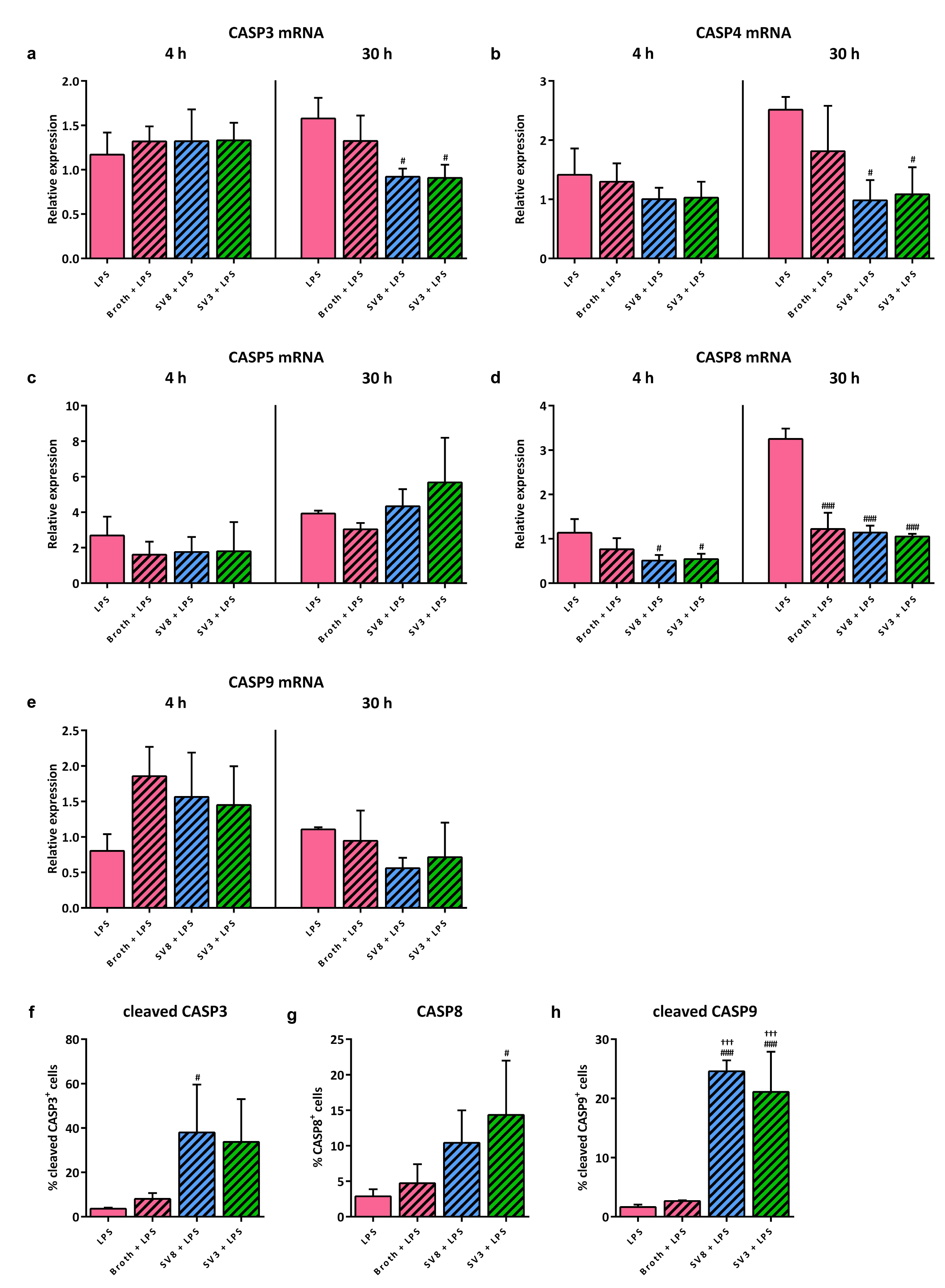

Supplement: S3 Fig — After 4 and 30 h of co-stimulation of A549 cells, caspase mRNA levels were assessed via qRT-PCR (a-e), and relative expression was calculated using the ΔΔCT method. Flow cytometry was used to determine caspase protein or activity after 24 h stimulation (f-h), the respective gating strategy is illustrated in S1 Fig. Data are shown as means ± SD and were obtained from n ≥ 3 independent experiments. # p < 0.05 and ### p < 0.001 compared to cells treated with LPS; ††† p < 0.001 compared to cells treated with broth+LPS. SV8: Ureaplasma urealyticum serovar 8, SV3: Ureaplasma parvum serovar 3. (TIF) [file pone.0216569.s003.tif]

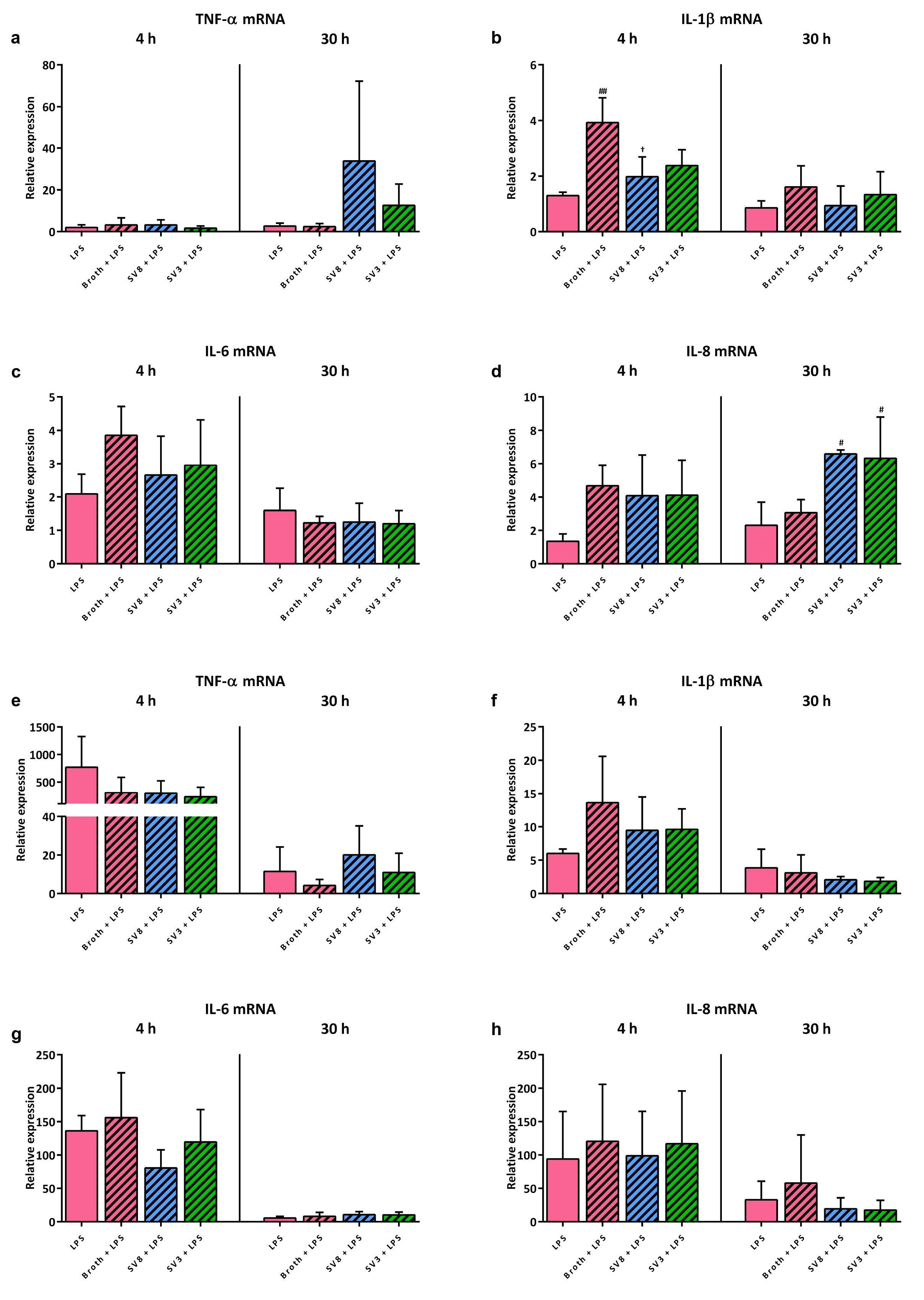

Supplement: S4 Fig — Cytokine mRNA levels were assessed via qRT-PCR in A549 cells (a-d) and HPMEC (e-h) following 4 and 30 h of co-stimulation. Data are presented as means ± SD from n ≥ 3 independent experiments. # p < 0.05 and ## p < 0.01 compared to cells treated with LPS; † p < 0.05 compared to cells treated with broth+LPS. SV8: Ureaplasma urealyticum serovar 8, SV3: Ureaplasma parvum serovar 3. (TIF) [file pone.0216569.s004.tif]
